# Biotechnological Production of the Cell Penetrating Antifungal PAF102 Peptide in *Pichia pastoris*

**DOI:** 10.3389/fmicb.2019.01472

**Published:** 2019-06-27

**Authors:** Crina Popa, Xiaoqing Shi, Tarik Ruiz, Pau Ferrer, María Coca

**Affiliations:** ^1^Centre for Research in Agricultural Genomics (CSIC-IRTA-UAB-UB), Barcelona, Spain; ^2^Department of Chemical, Biological and Environmental Engineering, Universitat Autònoma de Barcelona, Barcelona, Spain

**Keywords:** antimicrobial peptides, antifungal peptides, *Pichia pastoris*, plant oleosin, cecropin A, PAF peptides, lipid droplets

## Abstract

Antimicrobial peptides (AMPs) have potent and durable antimicrobial activity to a wide range of fungi and bacteria. The growing problem of drug-resistant pathogenic microorganisms, together with the lack of new effective compounds, has stimulated interest in developing AMPs as anti-infective molecules. PAF102 is an AMP that was rationally designed for improved antifungal properties. This cell penetrating peptide has potent and specific activity against major fungal pathogens. Cecropin A is a natural AMP with strong and fast lytic activity against bacterial and fungal pathogens, including multidrug resistant pathogens. Both peptides, PAF102 and Cecropin A, are alternative antibiotic compounds. However, their exploitation requires fast, cost-efficient production systems. Here, we developed an innovative system to produce AMPs in *Pichia pastoris* using the oleosin fusion technology. Oleosins are plant-specific proteins with a structural role in lipid droplet formation and stabilization, which are used as carriers for recombinant proteins to lipid droplets in plant-based production systems. This study reports the efficient production of PAF102 in *P. pastoris* when fused to the rice plant Oleosin 18, whereas no accumulation of Cecropin A was detected. The Ole18-PAF102 fusion protein targets the lipid droplets of the heterologous system where it accumulates to high levels. Interestingly, the production of this fusion protein induces the formation of lipid droplets in yeast cells, which can be additionally enhanced by the coexpression of a diacylglycerol transferase gene that allows a three-fold increase in the production of the fusion protein. Using this high producer strain, PAF102 reaches commercially relevant yields of up to 180 mg/l of yeast culture. Moreover, the accumulation of PAF102 in the yeast lipid droplets facilitates its downstream extraction and recovery by flotation on density gradients, with the recovered PAF102 being biologically active against pathogenic fungi. Our results demonstrate that plant oleosin fusion technology can be transferred to the well-established *P. pastoris* cell factory to produce the PAF102 antifungal peptide, and potentially other AMPs, for multiple applications in crop protection, food preservation and animal and human therapies.

## Introduction

Antimicrobial peptides (AMPs) are natural compounds with antimicrobial activity toward a wide range of fungi and bacteria. They are a diverse group of peptides with no consensus sequence associated to their biological activity but sharing common features: most of them are cationic, relatively hydrophobic and amphipathic molecules (Zasloff, 2002). Being amphipathic facilitates their selective interaction with negatively charged microbial membranes, so are capable of functioning as membrane-disruption peptides or cell-penetrating peptides interfering with key intracellular processes (Zasloff, 2002; Nicolas, 2009). In general, AMPs seem to act on low-affinity targets, so with little propensity to develop resistance in microbial targets (Zasloff, 2002; Hancock and Sahl, 2006; Peschel and Sahl, 2006; Sierra et al., 2017; Yu et al., 2018; Ghosh et al., 2019). The growing problem of resistance to conventional antibiotics together with the lack of new effective molecules has stimulated interest in developing AMPs as an alternative (Hancock and Sahl, 2006; Zhang and Gallo, 2016; Ghosh et al., 2019). In addition, AMPs have different mechanisms of action to other antimicrobials, reinforcing their enormous potential as a complement to conventional antibiotics. They have multiple applications in medical therapies, food preservation and crop protection (Marcos et al., 2008; López-García et al., 2012; Montesinos et al., 2012; Kang et al., 2017).

Although research on AMP development has been highly active recently, few molecules have entered the market. Major challenges to be solved before natural AMPs can be fully exploited relate to their target specificity and stability, as well as production costs. The advances in peptide synthesis have enabled the *de novo* synthesis and rational design of AMPs with improved properties (Marcos et al., 2008), with the hexapeptide PAF26 (RKKWFW) identified through a combinatorial approach as a novel peptide with specific antifungal activity and cell penetrating properties (López-García et al., 2002), and PAF102 rationally designed as a modified concatemer of PAF26 of 29 amino acid residues with high potency and specificity toward fungal cells, low toxicity to mammalian cells, high stability to proteolysis and for optimal production in plants (López-García et al., 2015). Other strategies are based on the modification of natural AMPs, such as the cecropin::melittin hybrids that are designed to decrease the toxicity of the high hemolytic melittin peptide (Cavallarin et al., 1998; Badosa et al., 2007, 2013). However, some naturally occurring peptides, such as the 37 residues peptide cecropin A (CecA) isolated from insects, have potent lytic activity against major bacterial and fungal pathogens without additional modifications (Steiner et al., 1981; Cavallarin et al., 1998; Moreno et al., 2003). More importantly, CecA has no lytic activity against mammalian erythrocytes or lymphocytes (Steiner et al., 1981; Suttmann et al., 2008). These properties make this natural peptide a good candidate to be developed as an antimicrobial in multiple fields of applications.

The other major limitation for most AMPs is their production costs. The amount of AMPs produced in living organisms is very low, and their extraction and purification requires complex and costly procedures. Chemical synthesis is only economically viable for short peptides and high value applications. Their peptidic nature enables production through biotechnological systems, but, depending on the nature of the AMPs, their production in conventional microbial systems is frequently not very efficient due to toxicity toward host cells or proteolysis of the products (Valore and Ganz, 1997; Bryksa et al., 2006; Jin et al., 2006; Wang et al., 2018). Recently, our group has demonstrated that rice seeds are good biofactories for linear AMPs that, otherwise, are difficult to produce in biological systems (Bundó et al., 2014, 2019; Montesinos et al., 2016, 2017). We found that, interestingly, PAF102 and CecA could be efficiently produced in rice seeds as oleosin fusion proteins targeted to oil bodies (OBs) (Montesinos et al., 2016; Bundó et al., 2019). OBs, also known as lipid droplets (LDs), are specialized structures composed mainly of a core of neutral lipids (triacylglycerols and steryl esters) surrounded by a monolayer of phospholipids containing a number of proteins that differ considerably between species (Chapman et al., 2012). Oleosins are plant specific proteins with a structural role in OB formation and stabilization (Tzen, 2012). They have been used as a carrier of recombinant proteins to OBs, facilitating their subsequent purification from plant material (van Rooijen and Moloney, 1995). Consequently, plant OBs have emerged as a target for biotechnological production of recombinant proteins (Parmenter et al., 1995; Nykiforuk et al., 2006, 2011; Boothe et al., 2010).

The purpose of the present study was to investigate whether oleosin fusion technology could be transferred to the well-established production system based on *Pichia pastoris* to efficiently produce AMPs. *P. pastoris* has been developed as an excellent host for the production of biopharmaceuticals and industrial enzymes. The growth of yeast is fast, with very high cell densities producing grams of recombinant protein per liter of culture (Ahmad et al., 2014). Here, PAF102 was efficiently produced in *P. pastoris* when fused to the rice plant Oleosin 18, whereas no accumulation of CecA was detected. The plant oleosin was correctly targeted to the LDs in the heterologous system and carries the PAF102 peptide, leading to high accumulation of the fusion protein in these vesicles. Moreover, the recombinant LDs were easily isolated by flotation on density gradients for PAF102 purification. We also show that the recovered PAF102 was biologically active against pathogenic fungi. Our results demonstrate that *P. pastoris* allows fast and efficient production of AMPs through oleosin fusion technology.

## Materials and Methods

### Plasmid Constructs, Yeast Strains and Growth Conditions

**FIGURE 1:**
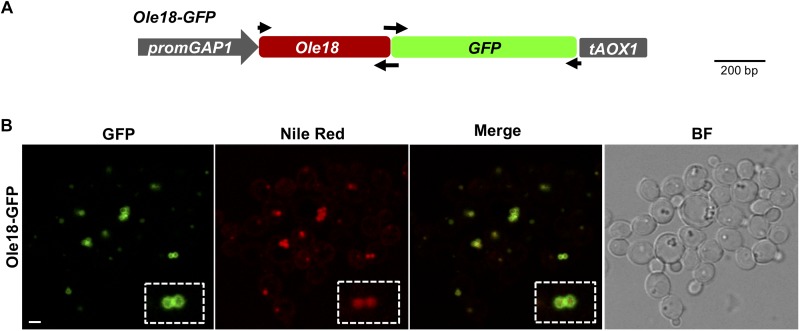
Ole18-GFP is targeted to *P. pastoris* LDs **(A)** Gene construct used to produce the Ole18-GFP fusion protein in *P. pastoris* cells. Expression of the fusion gene encoding the rice Ole18 protein tagged with the Green Fluorescent Protein (Ole18-GFP) was under the control of the constitutive promoter *GAP1* (promGAP1) and the *AOX1* terminator (tAOX1). Arrows indicate the position of primers used for preparing the construct **(B)** Confocal laser microscopy images of cells accumulating the Ole18-GFP protein visualized under fluorescence for GFP, Nile red, and the merged GFP and Nile red image. The corresponding bright field image (BF) is also shown. Scale bar = 2 μm.

A construct was prepared to produce the fusion protein of Oleosin 18 to the Green Fluorescent Protein (Ole18-GFP) in *P. pastoris* (Figure 1). The open reading frame of the rice *Ole18* (LOC_Os03g49190) without the stop codon was amplified by PCR from the plant vector pC::pOle18:Ole18-CecA (Montesinos et al., 2016) using the primers indicated in Supplementary Table 1 (EcoRI_Ole18fwd and Ole18rev) and the Phusion high-fidelity DNA polymerase (Thermo Scientific, Spain). The *GFP* open reading frame was amplified from the pYGWY yeast vector containing the GFP tag (Popa et al., 2016) using the GFPfwd and XbaI_GFPrev primers (Supplementary Table 1). Amplified DNA fragments were assembled by the Gibson reaction using the NEbuilder HiFi DNA assembly master mix (New England Biolabs, United States). The assembled *Ole18-GFP* fragment was then digested with the *EcoR*I and *Xba*I restriction enzymes, and cloned into these restriction sites of the integrative yeast vector pGAPHA for expression, driven by the Glyceraldehyde-3-Phosphate Dehydrogenase (*GAP*) constitutive promoter. This plasmid derives from pGAPZA (Invitrogen, Thermo Scientific, Spain), in which the Zeocin^TM^ resistance marker was replaced by the hygromycin resistance one (Adelantado, 2016).

**FIGURE 2:**
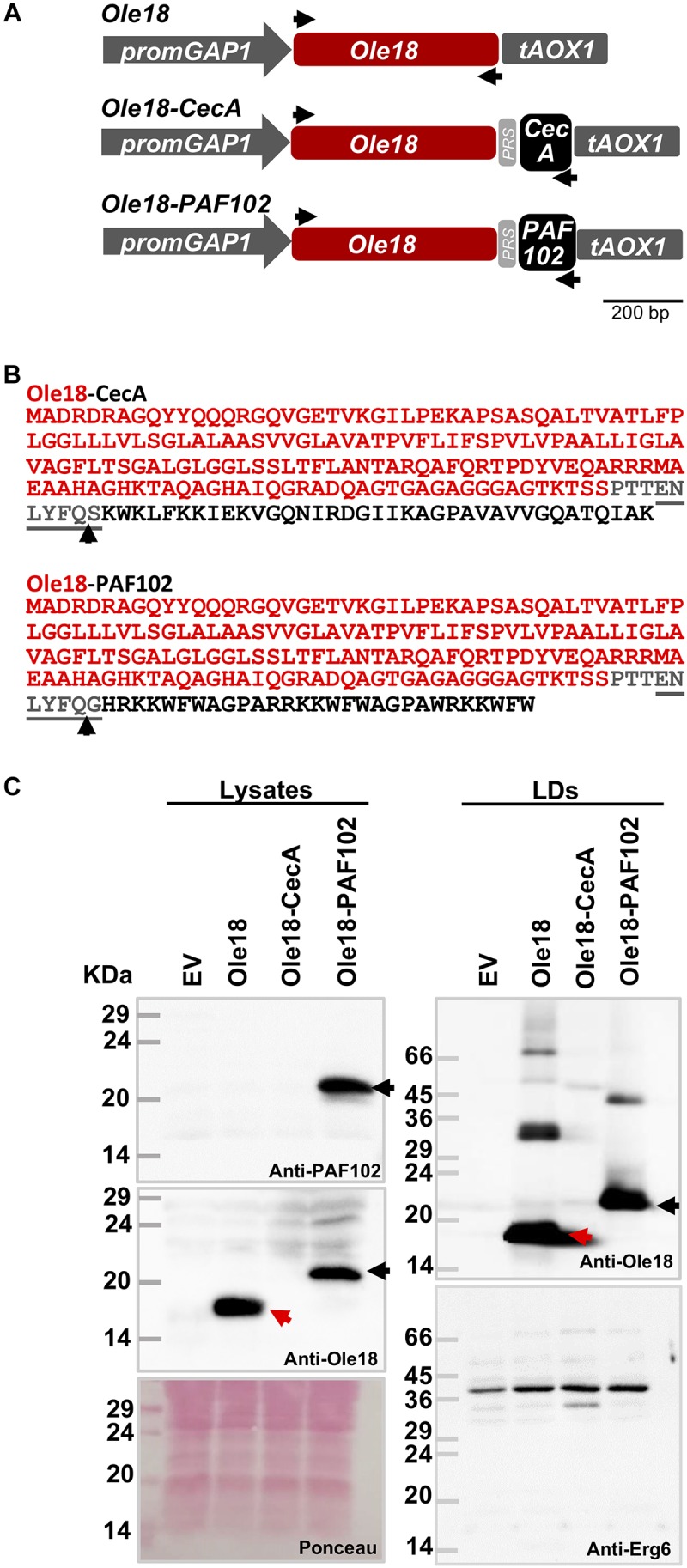
Ole18-PAF102 accumulates in *P. pastoris* LDs. **(A)** Gene constructs used to produce the rice Ole18 and the Ole18-CecA and Ole18-PAF102 fusion proteins in *P. pastoris cells*. Ole18 protein and the CecA and PAF102 antimicrobial peptides are linked through the TEV protease recognition site (PRS). Arrows indicate the position of primers used for preparing the constructs. **(B)** Amino acid sequence of the Ole18-CecA and Ole-PAF102 fusion proteins with Ole18 protein (red) linked through PRS (gray) to CecA or PAF102 peptides (black). Arrows indicate TEV protease cleavage site. **(C)** Immunoblot analysis of equivalent total protein extracts (lysates) and LDs from *P. pastoris* cell cultures transformed with empty vector (EV) or the indicated gene constructs, using anti-PAF102, anti-Ole18 or anti-Erg6 antibodies as indicated. Ponceau staining of the membrane is shown as protein loading control (25 μg of total protein). Red arrows indicate Ole18 protein bands and black the Ole18-PAF102 fusion protein bands. Molecular weight markers are indicated in kDa on the left.

Two more constructs were prepared to produce the Ole18-CecA and Ole18-PAF102 fusion proteins in *P. pastoris* (Figure 2). The *Ole18-CecA* open reading frame was amplified by PCR from the plant vector pC::pOle18:Ole18-CecA (Montesinos et al., 2016) using the EcoRI_Ole18fwd and XhoI_CecArev primers, shown in Supplementary Table 1. The *Ole18-PAF102* was amplified from the pC::pOle18:Ole18-PAF102 (Bundó et al., 2019) using the same forward primer and the XhoI_PAF102rev as the reverse primer (Supplementary Table 1). The fusion genes were introduced into the pGAPHA plasmid as *EcoR*I-*Xho*I fragments behind the *GAP* promoter. As a control, we also prepared a construct for the single Ole18 protein (Figure 2). For that, the *Ole18* open reading frame was amplified with EcoRI_Ole18fwd and XhoI_Ole18rev primers (Supplementary Table 1), and transferred to the pGAPHA plasmid as an *EcoR*I-*Xho*I fragment.

An additional construct was prepared for the production of Diacylglycerol O-AcylTransferase 1 (Dgat1) from *Arabidopsis thaliana* (At2g19450) in *P. pastoris*. In this case the open reading frame was amplified from the G16373 clone provided by the Arabidopsis Biological Resource Center (ABRC, Ohio State University, United States) using the primers Dgat1_fwd and Dgat1_rev (Supplementary Table 1). The amplified fragment was inserted into the pGAPZA plasmid (Invitrogen, Thermo Scientific, Spain) using the Gibson assembly reaction. This plasmid allows gene expression under the control of the *GAP* promoter and confers Zeocin^TM^ resistance. All the constructs were verified by DNA sequencing.

Gene constructs were transferred to the wild-type *P. pastoris* strain X-33 by electroporation. Recombinant colonies were isolated using the appropriate antibiotic selection at 100 μg/ml of Zeocin^TM^ or 300 μg/ml of hygromycyn B, and then confirmed by PCR. Two to three independent colonies were analyzed by transformation event with similar results in the different assays. Yeast cells were grown in YPD medium (1% yeast extract, 2% peptone, and 2% glucose) at 30°C. Cell growth was monitored by measuring the optical density at 600 nm (OD_600_) of the culture at different time points, or by plating serial 1/10 dilutions of an exponential growing culture on YPD agar plates.

### Lipid Droplets Fractionation

Lipid droplets (LDs), along with their bound proteins, were isolated by density gradient centrifugation provided by Ficoll (Sigma-Aldrich, Spain), as previously described, with some modifications (Mannik et al., 2014). Basically, the method was adapted to lyse the cells by sonication on ice. Cells grown in YPD medium to late logarithmic phase were resuspended in Ficoll buffer I (12% Ficoll, 10 mM Tris–Hcl, 200 μM EDTA) at a concentration of 2 ml/g of cells. Cells were then lysed through seven consecutive cycles of sonication of 20 s each. After clarifying the lysate from the cell debris by low speed centrifugation, the supernatant was overlaid with the same volume of Ficoll buffer I and centrifuged at high speed (100,000 *g*) for 1 h in a SW40 rotor. LDs were recovered in the floating white layer, and then, were overlaid with a volume of Ficoll buffer II (8% Ficoll, 10 mM Tris–HCl, 200 μM EDTA). After a second round of flotation-centrifugation under the same conditions, LDs were obtained at high purity in the floating fat pad. The purity and integrity of recovered LDs were confirmed by selective staining with Nile red (1 ng/ml, Sigma-Aldrich, Spain) and fluorescence microscopy visualization.

### Immunoblot Analysis of LD-Associated Proteins and PAF102 Purification

LD-associated proteins were solubilized in loading buffer, separated by SDS-PAGE (15% acrylamide, Laemmli, 1970), transferred to nitrocellulose membranes and immunodetected with different antibodies: anti-Ole18 (1:2000 dilution, Montesinos et al., 2016), anti-CecA (1:500 dilution, Coca et al., 2006), anti-PAF102 (1:1000 dilution, Bundó et al., 2019), and anti-Erg6 (1:500 dilution, Paltauf et al., 1994). PAF102 accumulation was estimated on immunoblot by quantification of signal intensities of Ole18-PAF102 to known amounts of synthetic PAF102, using the Quantity Tools Image Lab^TM^ Software (Version 5.2.1) included in the ChemiDoc^TM^ Touch Imaging System (Bio-Rad, United States).

OBs containing Ole18-CecA or Ole18-PAF102 proteins were isolated from *pOle18:Ole18-CecA* and *pOle18:Ole18-PAF102* rice seeds as previously described (Montesinos et al., 2016; Bundó et al., 2019). OB fractions obtained from ten seeds and resuspended in 100 μl buffer (0.2 M sucrose, 10 mM phosphate buffer pH 7.6) were separated into four aliquots (25 μl each). Protease inhibitor cocktail (Sigma-Aldrich, Spain) was added to two of these, and then 25 μg of total protein extracts from the empty vector *P. pastoris* strain was added to all them. One with and one without the inhibitor were immediately frozen and the other two incubated at room temperature overnight. Proteins were then analyzed by immunoblot using anti-Ole18 specific antibodies.

PAF102 was recovered from the LDs containing the Ole18-PAF102 fusion protein by digestion with Tobacco Etch Virus (TEV) protease (Invitrogen, Thermo Fisher Scientific, Spain, 1:100), as the Ole18 protein was linked to the PAF102 peptide through a TEV protease recognition site (ENLYFQ/G). Proteolytic digestion was overnight at 30°C in TEV protease buffer. The estimated efficiency of the TEV protease was based on the disappearance of the Ole18-PAF102 signal by immunoblot analysis. The released PAF102 was immunodetected using anti-PAF102 antibodies by western-blot, after acetone precipitation, separation in Tricine-SDS-PAGE (18% acrylamide, Schägger and von Jagow, 1987) and transfer to nitrocellulose membranes (Whatman^®^ Potran 0.2 μm).

### Confocal and Fluorescence Microscopy

Cells of *P. pastoris* carrying the different gene constructs were visualized under confocal fluorescent microscopy with a Leica TCS SP5 microscope. For that, cells grown overnight in liquid medium to late logarithmic phase (1 ml of cultures) were concentrated by centrifugation for 2 min at 600 *g*, washed in water, resuspended in phosphate-buffered saline (PBS) containing 1 ng/ml of Nile red and directly visualized under a coverslip. GFP signal was detected at 500–550 nm through excitation with an Argon ion laser emitting a 488 nm and Nile red signal at 570–670 nm using DPSS laser emitting at 561 nm.

Cell viability was evaluated by fluorescein diacetate (FDA, Sigma-Aldrich, Spain) staining (Jones and Senft, 1985). Cell cultures grown at logarithmic phase (OD_600_ = 1) were concentrated, washed and resuspended in PBS containing FDA (10 μg/ml) and stained for 20 min. After washing, the cells were visualized under fluorescent microscopy using an Axiophot Zeiss upright wide field fluorescence microscope.

The size of the intracellular LDs was estimated by image analysis using ImageJ software. The Z-projection fluorescent images were converted to binary images by applying a threshold that highlights all the structures to be measured. The area of LDs was determined using the particle analysis tool and taking into account only those droplets with spherical geometry. The 100 LDs measured per line were clustered in different size ranges and the percentage of LDs in each group was calculated. To determine the amount of LDs in the different cell lines, LDs were counted in three different replicas of the projection images of 30 cells from at least three different experiments.

### Antifungal Activity Assays

Growth inhibition assays of *Fusarium proliferatum* were in 96-well flat-bottom microtiter plates as previously described, with minor changes (López-García et al., 2015). Basically, 50 μl of fungal conidia (5 × 10^4^ conidia/ml), in 1/10 diluted potato dextrose broth (PDB) containing 0.02% (w/v) chloramphenicol, were mixed in each well with 50 μl of LD fractions. The Ficoll buffer II of LD fractions was exchanged with sterile deionized H_2_O containing 0.02% Tween-20 using Zeba^TM^ Spin Desalting columns (Thermo Fisher Scientific, Spain). Samples were prepared in triplicate. Plates were incubated with agitation for 72 h at 28°C. Fungal growth was monitored every 24 h by measuring the OD_600_ using a Spectramax M3 reader (Molecular Devices, United States), and mean values and standard deviation (SD) were calculated. Experiments were repeated twice with similar results.

## Results

### Ole18-GFP Fusion Protein Is Targeted to *P. pastoris* LDs

A gene construct was prepared to study the subcellular localization of the rice Ole18 protein in *P. pastoris* (Figure 1A). This construct was designed to express a *Ole18-GFP* fusion gene under the control of the constitutive promoter *GAP1* in the integrative yeast vector pGAPHA. Cell colonies carrying the gene construct were obtained by genetic transformation. Their growth rate in rich medium was similar to cells transformed with the empty vector (EV). Confocal fluorescence microscopy revealed the GFP signal surrounding spherical intracellular vesicles with diameters of 0.1–1 μm (Figure 1B). These vesicles co-localized with those labeled with Nile red, a fluorescent stain for neutral lipids used as an LD marker (Greenspan et al., 1985). These results indicate that the Ole18-GFP fusion protein is produced and accumulates mostly in the periphery of LDs of *P. pastoris* cells. Hence, the rice Ole18 protein was efficiently produced and correctly targeted to LDs in this heterologous system, as previously reported for other plant oleosin proteins produced in *Saccharomyces cerevisiae* yeast cells (Ting et al., 1997; Beaudoin et al., 2000; Parthibane et al., 2012; Jacquier et al., 2013; Vindigni et al., 2013). Our results also suggest that Ole18 can be used as a carrier protein to LDs in Pichia cells.

### Ole18-PAF102 Accumulates in *P. pastoris* LDs

To investigate whether the oleosin fusion technology can be used to produce AMPs in *P. pastoris*, we prepared two new gene constructs (Figure 2A). They were designed to produce the CecA and PAF102 antimicrobial peptides as fusion to the rice Ole18 protein (Figure 2B). Proteins were linked through a TEV protease recognition site for the subsequent release of the AMPs from the fusion protein. We also prepared an additional construct to produce the single Ole18 protein as a control (Figure 2A). Transformed colonies were obtained for each construct, and integration of the transgenes was confirmed by PCR. Total protein extracts from the recombinant cultures grown in YPD media to the late logarithmic phase were analyzed by SDS-PAGE and immunoblotting (Figure 2C, right panels). Using specific antibodies to the rice Ole18, we detected a band of the expected size (around 18 kDa) in the lysates obtained from *Ole18* strain cultures (Figure 2C, red arrows). A higher band was detected in the lysates from *Ole18-PAF102* cultures (Figure 2C, black arrows) with an apparent size around 23 kDa, the expected size for the Ole18-PAF102 fusion protein (the theoretical molecular weight is 22.17 kDa). This band was also immunodetected using the anti-PAF102 specific antibodies. Our results indicate that the Ole18-PAF102 fusion protein was produced and stably accumulated in *P. pastoris* cells.

In contrast, no immunoreactive band was detected in the protein extracts from the *Ole18-CecA* cultures, using anti-Ole18 (Figure 2C) or anti-CecA antibodies (data not shown). The theoretical molecular weight of Ole18-CecA fusion proteins is 22.32 kDa. Up to five independent *Ole18-CecA* transformed colonies confirmed for transgene integration were tested, with similar results. This data suggests that the Ole18-CecA fusion protein did not accumulate at detectable levels in the yeast cells.

We then isolated the LDs from the different recombinant yeast strains by gradient density centrifugation. LD fractions were stained with Nile red and visualized under fluorescence and bright field microscopy. Samples had a high content of intact, high purity LDs. After solubilizing in SDS loading buffer, the LD proteins were separated by SDS-PAGE and immunodetected using specific anti-Ole18 antibodies (Figure 2C, right upper panel). We clearly detected the presence of Ole18 (red arrow) and Ole18-PAF102 (black arrow) proteins in the LD fractions, but not the Ole18-CecA protein (Figure 2C, right upper panel). The isolated fractions were additionally confirmed as LD fractions by immunodetection of the Erg6 protein (Figure 2C, right lower panel), an enzyme of the ergosterol biosynthetic pathway known to be associated with the LDs in *P. pastoris* cells (Ivashov et al., 2013). These results show that Ole18-PAF102 accumulates in *P. pastoris* LDs.

### Ole18-CecA Is Susceptible to *P. pastoris* Proteases

**FIGURE 3:**
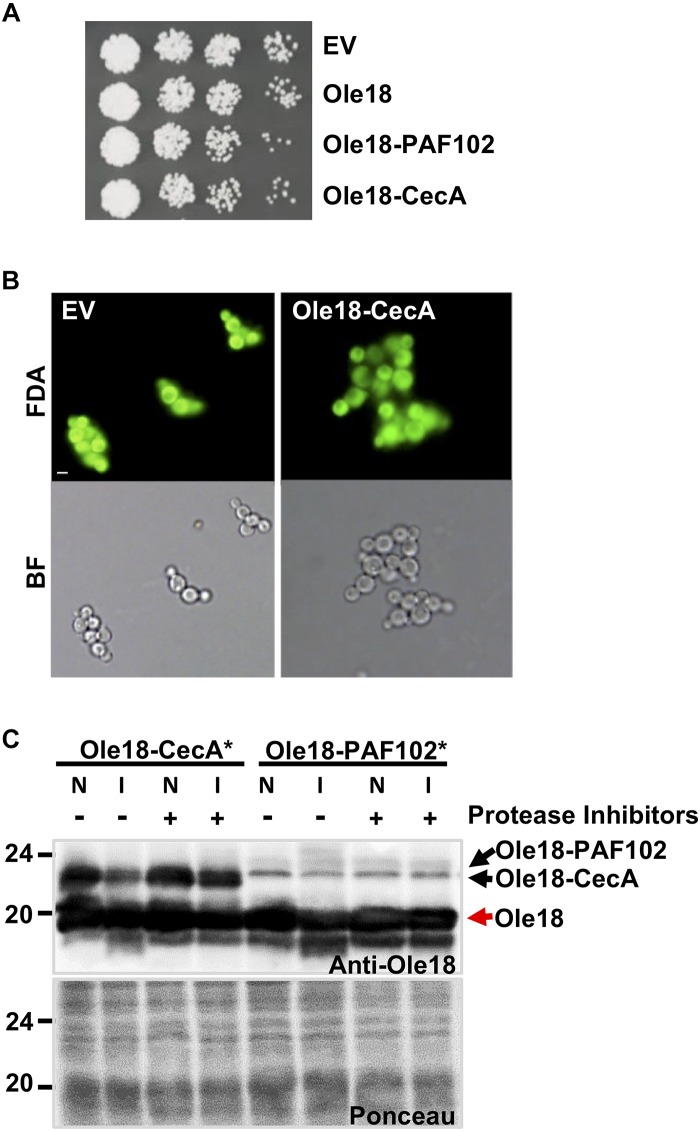
Ole18-CecA is susceptible to *P. pastoris* proteases. **(A)** Growth of recombinant strains carrying empty vector (EV), *Olel8, Olel8-PAF102, Olel8-CecA.* Aliquots of 5 μl of ten serial dilutions of log phase cultures (OD_600_ = 1) were spotted on YPD agar media and allowed to grow for 2 days at 30°C. **(B)** Images of fluorescence and bright field microscopy of EV and *Olel8-CecA* cells after fluorescein diacetate (FDA) viability staining. **(C)** Immunoblot of OB fractions isolated from rice seeds producing Ole18-CecA^∗^ or Ole18-PAF102^∗^ fusion proteins, in the presence of total protein extracts (25 μg) from EV yeast cells, supplemented without (–) or with (+) protease inhibitors, incubated (I) or not (N) overnight at room temperature.

Given that Ole18-CecA was not detected in total or LD protein extracts from the recombinant yeast strain, we hypothesized a potential toxicity problem related to the CecA antimicrobial activity, and this would lead to growth defects. However, when the growth of *Ole18-CecA* cells was monitored, we found they grew similarly to the strains transformed with the EV, the *Ole18*, and the *Ole18-PAF102* gene constructs (Figure 3A). Additionally, the morphological appearance and viability of *Ole18-CecA* cells was similar to EV transformed cells when visualized under fluorescence microscopy after fluorescein diacetate viable cell staining (Figure 3B). We found no evidence of toxicity associated to the *Ole18-CecA* gene in our experiments.

Another possible scenario could be that Ole18-CecA does not accumulate due to instability in the yeast cells. To test the stability, we obtained the fusion protein from *promOle18:Ole18-CecA* rice seeds previously generated in our group (Montesinos et al., 2016). These rice seeds accumulate large amounts of Ole18-CecA protein in OBs (40 μg per gram of rice seeds). Isolated rice OBs containing Ole18-CecA protein were incubated *in vitro* with *P. pastoris* protein extracts from EV strain, and then analyzed by immunoblotting (Figure 3C). There was a clear reduction in the amount of Ole18-CecA after incubation with Pichia protein extracts unless protease inhibitors were added. As a control, we also analyzed the Ole18-PAF102 fusion protein produced in rice seeds (Bundó et al., 2019). Interestingly, Ole18-PAF102 remained stable after overnight incubation with the Pichia protein extracts. These results suggest that Ole18-CecA does not stably accumulate in *P. pastoris* cells because it is susceptible to the yeast proteases, whereas Ole18-PAF102 is more resistant to yeast proteases (Figure 3C) and accumulates to high levels in the yeast system (Figure 2C). In contrast, accumulation of Ole18-CecA is higher than Ole18-PAF102 in rice (Figure 3C), probably associated to a higher susceptibility of Ole18-PAF102 to rice proteases. Therefore, *P. pastoris* seems to be an efficient system for the production of Ole18-PAF102, and hereinafter we focus on this antifungal peptide.

### Ole18-PAF102 Production Induces Proliferation of LDs in *P. pastoris*

**FIGURE 4:**
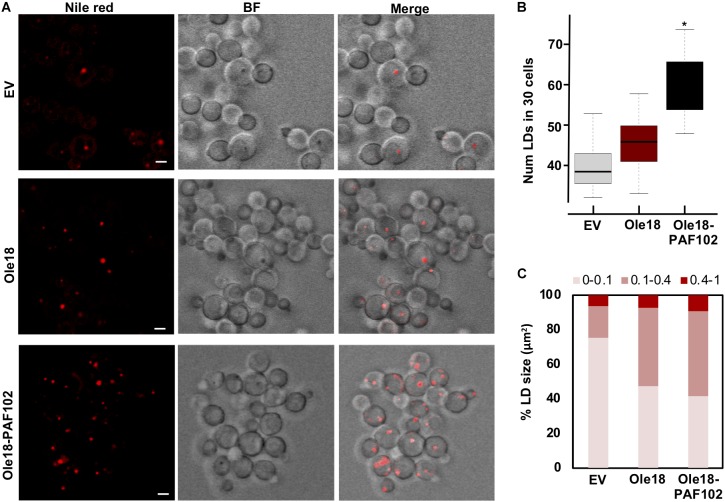
Ole18-PAF102 production induces proliferation of LDs in *P. pastoris* cells **(A)** Confocal microscopy images of EV, Ole18, and Ole18-PAF102 recombinant cells at late log growth phase stained with Nile red. Bright field (BF) images, and fluorescence and BF merged images are also shown. Images correspond to single focal sections. Bars = 2 μm. **(B)** Total number of LDs per 30 cells of the indicated strains. LDs were counted in two replicates per assays and five independent assays (*n* = 10). Asterisk denotes statistically significant differences (Tukey test, *p* < 0.005) **(C)** Distribution of range sizes (area in μm^2^) from 50 LDs from cells of indicated strains.

To further characterize the production of Ole18-PAF102 in the yeast cells, we closely inspected the recombinant cells under confocal microscopy (Figure 4A). We observed that their morphological appearance was similar to *EV* and *Ole18* cells, but they contained a higher number of LDs, with the cell-to-cell LD number and size highly variable. As the number of LDs was difficult to quantify at the cell level, we used a large population (30 cells), detecting a statistically significant increase in the cells accumulating Ole18-PAF102 (Figure 4B). Although there was also a tendency for a higher number of LDs in *Ole18* cells, no significant differences were quantified in comparison to EV cells. These results differ from reports in the literature showing an induction of LD proliferation mediated by other plant oleosins in the yeast *S. cerevisae* (Ting et al., 1997; Jacquier et al., 2013; Jamme et al., 2013; Vindigni et al., 2013). We also observed an increase in the size of the recombinant LDs, more pronounced in those of Ole18-PAF102 (Figure 4C). Our results suggest that the antifungal PAF102 peptide fused to Ole18 determines an increase in LDs in *P. pastoris* cells.

### *A. thaliana* *Dgat1* Expression Induces LD Formation in *Ole18-PAF102* Yeast Cells

**FIGURE 5:**
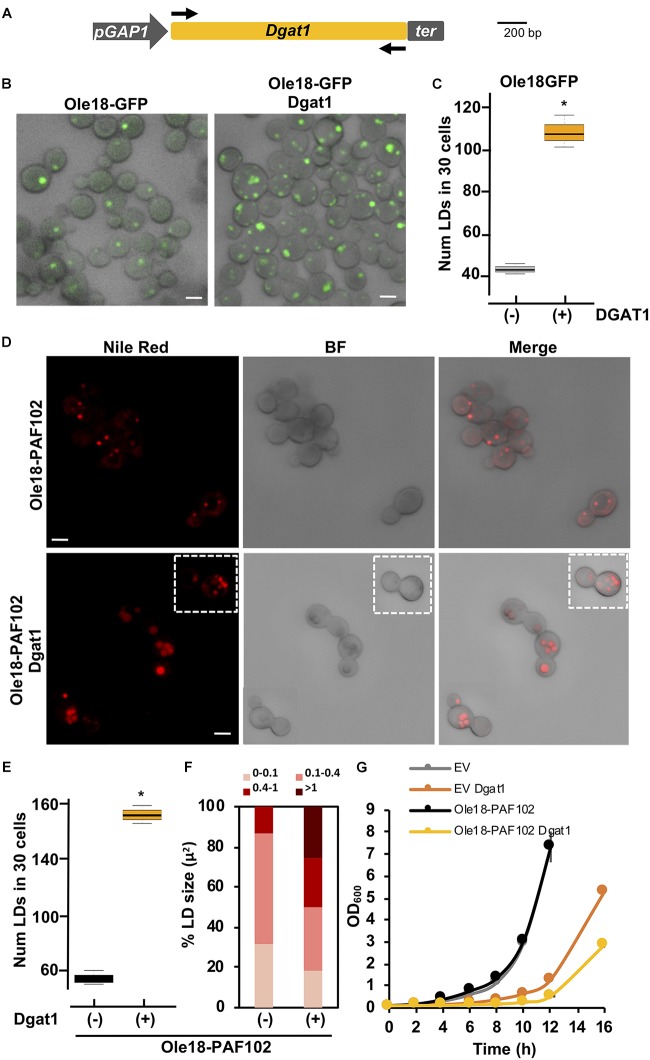
*A. thaliana* Dgat1 expression induces LD formation in Ole18-PAF102 yeast cells **(A)** Gene construct introduced into the pGAPZA vector for producing *A. thaliana* Diacylglycerol o-acyltransferase (Dgat1) enzyme (At2g19450) in *P. pastoris*. **(B)** Confocal microscopy images of Ole18-GFP cells with or without the Dgat1 construct, as indicated. Bright field (BF) and fluorescence merged images are shown. **(C)** Total number of LDs per 30 cells of the indicated strains. LDs were counted in three replicates. Asterisk denotes statistically significant differences (Tukey test, *p* < 0.005) **(D)** Confocal microscopy images of Nile red stained Ole18-PAF102 cells with or without the Dgat1 construct as indicated. Bright field (BF), and fluorescence and BF merged images are also shown. Images correspond to single focal sections. **(E)** Total number of LDs per 30 cells of the indicated strains. LDs were counted in three replicates. Asterisk denotes statistically significant differences (Tukey test, *p* < 0.005) **(F)** Distribution of range sizes (area in μm^2^) from 50 LDs from strain cells indicated. **(G)** Growth of indicated strain cultures. Graph represents the mean OD_600_ ± SD value of triplicate cultures measured every 2 h. Bars = 2 μm.

With the purpose to increase the amount of LDs and presumably the accumulation of Ole18-PAF102 in the yeast cells, we expressed the *Dgat1* gene from *A. thaliana* in *P. pastoris* cells. Dgat enzymes catalyze the acyl-CoA-dependent acylation of sn-1,2-diacylglycerol to produce triacylglycerol (TAG) (Liu et al., 2012). This is the rate-limiting activity for TAG biosynthesis and a target for engineering increase in oil content in microorganisms (Liu et al., 2012). The expression of the Arabidopsis *Dgat1* in *S. cerevisiae* has been reported to enhance the content in TAG and LD formation (Bouvier-Navé et al., 2000; Aymé et al., 2014). We prepared a new construct containing the coding sequence of the *AtDgat1* in the integrative pGAPZA vector, which carries another selection marker to allow double transformant selection (Figure 5A). To easily monitor the LD content, *Dgat1* transformation was first examined in the *Ole18-GFP* strain. Visualization of cells under confocal microscopy revealed an increase in the content of LDs in the recovered transformant cells (Figure 5B). Although there was a large variability among cells, most of *Ole18-GFP* possessed one or two LDs whereas the double transformant *Ole18-GFP*/*Dgat1* had three or four LDs per cell. The number of LDs was significantly higher in those cells expressing the *Dgat1* gene (Figure 5C), indicating that expression of the Arabidopsis *Dgat1* gene induces LD formation in *P. pastoris*, as reported for *S. cerevisiae* (Bouvier-Navé et al., 2000; Aymé et al., 2014).

We then introduced the *Dgat1* gene construct in the *Ole18-PAF102* strain. Double transformants were recovered and visualized under confocal microscopy. Nile red staining of LDs revealed accumulation of cellular LDs mediated by the expression of the *Dgat1* gene (Figure 5D). We found a statistically significant increase of around three times in the number of LDs of double transformant cells (Figure 5E), which not only had more LDs, but were also larger in size (Figure 5F). Consistent with our previous observations, there was high variability in the number and size of LDs in the cell population. Monitoring cell growth through optical density (OD_600_), we also found that cells expressing *Dgat1* were slower reaching high densities (Figure 5G). This slow growth was not only measured in the *Ole18-PAF102* strain but also in *EV* cells, and both EV and *Ole18-PAF102* single transformants had similar growth rates. This indicates that the delay may be determined by *Dgat1* gene expression. Altogether, our results show that the LD content is further enhanced in *Ole18-PAF102* strain by expression of the Arabidopsis *Dgat1* gene, albeit requiring a longer period of culture growth.

### Biologically Active PAF102 Is Recovered From Yeast Cells

**FIGURE 6:**
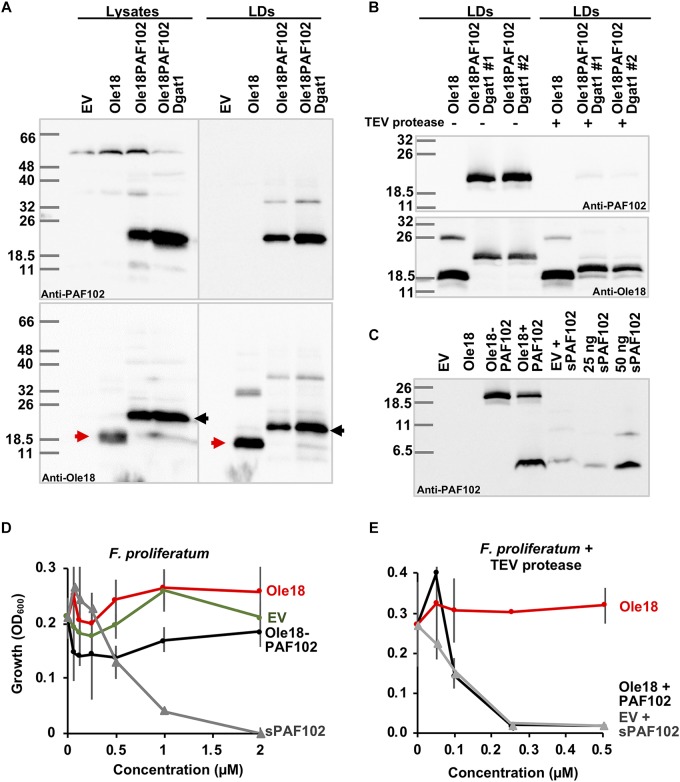
Biologically active PAF102 is recovered from yeast cells. **(A)** Immunoblot of total protein extracts (25 μg) and LD proteins from *P. pastoris* cell cultures transformed with empty vector (EV) or the indicated gene constructs, using anti-Ole18 or anti-PAF102 antibodies as indicated. **(B)** Immunoblot analysis of LDs fractions before (–) or after (+) TEV protease digestion using anti-PAF102 or anti-Ole18 antibodies as indicated. LDs were from Ole18 and Ole18PAF102/Dgat1 (#1 and #2 independent colonies) strains. **(C)** Immunoblot analysis of PAF102 peptide released from the fusion Ole18-PAF102 protein. Proteins from LDs were precipitated with acetone after TEV protease digestion and separated in Tricine-SDS-PAGE. Synthetic PAF102 (sPAF102, 25 or 50 ng), alone or in combination with the EV proteins, was run in parallel for comparative purposes **(D,E)** *F. proliferatum* growth inhibitory assays by indicated LD fractions in comparison to synthetic PAF102 (sPAF102) at varying concentrations **(D)** or by TEV digested LDs fractions in comparison to EV LDs supplemented with sPAF102 **(E)**.

Knowing that double transformant *Ole18-PAF102/Dgat1* cells contain a high number of LDs, and that Ole18-PAF102 is accumulated in these LDs, we evaluated the amount of PAF102 peptide produced by this strain. We isolated the LDs by density gradient centrifugation and analyzed their Ole18-PAF102 content by immunodetection. As shown in Figure 6A, Ole18-PAF102 accumulation was higher in the *Dgat1* expressing strain and correctly targeted to LDs. We estimated that the fusion protein accumulates at 67 ± 2 mg/l in the *Ole18-PAF102* strain, and reaches up to 180 ± 5 mg/l in the *Ole18-PAF102/Dgat1* strain. This represents a near three-fold increase in the fusion protein accumulation levels, congruently with the three-fold increase in cellular LD content (Figure 5E).

Using this high producer *Ole18-PAF102/Dgat1* strain, we assessed the recovery of the single PAF102 peptide from the LDs containing the Ole18-PAF102 fusion protein. TEV protease was used to digest the recombinant LD fraction since the Ole18 and PAF102 polypeptides were linked through the TEV protease recognition site (PRS). In immunoblot analysis of LD fractions after proteolytic digestion (Figure 6B), we observed that the fusion protein detected by anti-PAF102 antibodies was almost absent (upper panel), and a protein slightly larger than Ole18 appeared, recognized by anti-Ole18 antibodies (lower panel). After TEV protease digestion, most of the PRS remained attached to the Ole18 protein. This gives the Ole18 released from the fusion protein by TEV protease digestion a theoretical molecular weight of 18.25 kDa, whereas the single Ole18 is only 17.15 kDa. The bands detected in western-blot analysis are consistent with this difference in size and suggest that the fusion protein was processed by the TEV protease. Based on the disappearance of the fusion protein in different assays, the TEV protease efficiency was calculated at 86% ± 10.5 on average. This data indicates a high efficiency of proteolytic processing of the fusion protein Ole18-PAF102 on intact LDs. We then investigated the presence of the PAF102 single peptide in the protease digested fractions. We immunodetected a polypeptide in the Ole18PAF102 LDs with similar mobility to the synthetic PAF102 (sPAF102) added to the EV LDs, or to the sPAF102 alone (Figure 6C). This band was not present in Ole18 LDs or in non-digested Ole18PAF102 LDs, indicating that the fusion protein was correctly processed and the PAF102 released.

To characterize the released PAF102 peptide, we tested the antifungal activity of the LDs fractions before and after TEV protease digestion. For that, we monitored the growth of *F. proliferatum* in the presence of LDs at different concentrations. We did not detect any fungal growth inhibitory activity in the intact LD fractions, regardless of whether they were from EV, *Ole18* or *Ole18-PAF102* strains (Figure 6D), whereas the sPAF102 completely inhibited *F. proliferatum* growth at the very low concentration of 2 μM. This data indicates that the fusion protein Ole18-PAF102 was not active, at least when embedded into LDs. However, TEV protease digested Ole18-PAF102 LDs clearly inhibited the growth of *F. proliferatum*, and the inhibitory activity was equivalent to that of EV LDs supplemented with sPAF102 and incubated with TEV protease (Figure 6E). It is worth noting that the synthetic PAF102 was more active in the presence of EV LDs and TEV protease, completely inhibiting fungal growth at the even lower concentration of 0.25 μM. Given that Ole18 LDs and TEV protease did not have any inhibitory activity (Figure 6E), TEV protease could be assisting PAF102 activity. Our results demonstrate that when PAF102 is produced in *P. pastoris* using the oleosin fusion technology, it is biologically active and equivalent to that produced by chemical synthesis.

## Discussion

This study reports that *P. pastoris* is an excellent cell factory for the fast and efficient biotechnological production of the antifungal PAF102 peptide, and probably other PAF peptides. The process developed here is an innovative system based on the plant oleosin fusion technology that we successfully transfer to the heterologous yeast system. It has so far been difficult to produce rationally designed PAF antifungal peptides in conventional microbial expression systems, probably due to toxicity toward host cells. We recently demonstrated that these bioactive peptides could be produced in plant biofactories as oleosin fusion proteins (Bundó et al., 2019). The fusion of PAF102 to the rice Ole18 protein reduces its toxicity to the host cells allowing its accumulation to large amounts. We show here that the Ole18-PAF102 fusion protein is also produced in yeast cells and targeted to LDs where large amounts accumulate. By coexpressing the Arabidopsis *Dgat1* gene, *P. pastoris* cells produced up to 180 mg/l of PAF102 peptide in only 4 days. This yield was obtained from shake-flash cultures, and typically recombinant protein yield can be increased in fermenter cultures. This demonstrates that our new system is a considerable improvement on the previous rice-seed based system in terms of production times and yields of commercial relevance.

Nevertheless, the natural CecA peptide is not produced at detectable levels in this *P. pastoris* system, although it was efficiently produced in rice seeds when fused to Ole18, even to higher levels than PAF102 (Montesinos et al., 2016). Recombinant protein production is not only dependent on gene expression and protein synthesis but also on its stability in host cells to allow its accumulation to significant amounts. Therefore, the lack of Ole18-CecA accumulation to detectable levels by immunoblot analysis might be due to instability in *P. pastoris* cells. Indeed, our results show that CecA is highly susceptible to the yeast proteases. In contrast, PAF102 seems to be more resistant to Pichia proteases and accumulates to high levels. Reports have been already published on the high susceptibility to proteolytic degradation of CecA by fungal proteases (Bland and Lucca, 1998) or of CecA-derived peptides by tobacco plant proteases (Cavallarin et al., 1998). However, CecA stably accumulated in transgenic rice plants, suggesting limited proteolysis by rice proteases (Coca et al., 2006; Bundó et al., 2014; Montesinos et al., 2016). This data indicates differential degradation of CecA by host proteases, with its stability varying from one system to another: the stability of CecA to protein extracts derived from the host system should first be tested when planning to produce CecA in a new system. Still, the use of protease-deficient strains might improve production of CecA in *P. pastoris*. These strains have been used for the production of protease-sensitive proteins with variable success depending on the proteases involved in the degradation events (Ahmad et al., 2014). Our results suggest that the efficiency of the production system here developed would be dependent on the peptide to be produced.

Oleosin proteins have an important structural role in facilitating the formation and stability of plant LDs. They are small lipophilic proteins with a conserved central hydrophobic domain, inserted within the phospholipid bilayer of LDs, and two variable amphipathic N and C terminal domains covering the LD surface (Abell et al., 1997). Oleosins have an intrinsic affinity to membranes containing neutral lipids, associating spontaneously with them and assisting their sequestration into domains and ultimately in the formation of a particle filled with neutral lipids. Yeast LDs do not have similar structural proteins, but perform their functions properly (Koch et al., 2014). However, oleosins from different plant species produced in *S. cerevisiae* cells target LDs and induce the formation of LDs (Ting et al., 1997; Jacquier et al., 2013; Jamme et al., 2013; Vindigni et al., 2013). In agreement with these reports, rice Ole18 targets and stably accumulates in LDs in *P. pastoris*. The fusion of PAF102 to the Ole18 in the C-terminal also targets LDs, with the hydrophobic domain of Ole18 embedding into the TAG matrix and the PAF102 remaining on the surface. There were more and larger LDs in the cells accumulating the Ole18-PAF102 fusion protein than in those accumulating only the Ole18. It seems that the presence of PAF102 stabilizes the recombinant LDs and promotes their formation, probably due to the cationic and amphipathic character of PAF102 and its phospholipid affinity. This suggests that the oleosin fusion technology could be successfully applied to other AMPs with a similar cationic and amphipathic character.

A valuable addition to the production system is the coexpression of *AtDagt1* and *Ole18-PAF102* genes. *AtDag1* expression enhances the TAG content in yeast cells (Bouvier-Navé et al., 2000; Aymé et al., 2014). This high content of TAG leads to overaccumulation of the Ole18-PAF102 fusion protein, resulting in high yields of the recombinant protein. This is consistent with the requirement of TAGs for the stable accumulation of the *A. thaliana* Oleosin 1 in yeast cells (Jacquier et al., 2013). Oleosin 1 was easily degraded in mutant strains of *S. cerevisae* deficient in TAGs that were not able to produce and to accumulate neutral lipids. In contrast, cells with a high content of TAGs offer sufficient anchoring surface for the Ole18-PAF102, providing stability and allowing the accumulation of very large amounts of the fusion protein. The yield could be further improved by using modified *Dagt1* genes to enhance the storage lipid content in yeast (Greer et al., 2015).

One of the main advantages of the oleosin fusion technology is the simplicity of extraction and purification of the recombinant proteins accumulated in LDs from plant material. Similarly, *P. pastoris* LDs with the Ole18-PAF102 can be separated from other cellular components by simple flotation in density gradients. The yeast isolated LDs containing the Ole18-PAF102 had no activity against fungal targets, consistent with previous results with LDs from rice seeds (Bundó et al., 2019). PAF102 is not active while immobilized in the LDs, requiring release from the Ole18 for activity. We speculate that this cell penetrating peptide cannot enter the fungal target cell while attached to LDs, a process that is necessary for its antifungal action (Muñoz et al., 2013). Once released from Ole18, PAF102 produced in *P. pastoris* was biologically active and indistinguishable from the synthetic peptide. PAF102 has potent activity against *F. proliferatum*, a plant pathogen that contaminates cereal grain with mycotoxins, reducing grain quality and causing severe economic losses (Vismer et al., 2019). Here we demonstrate that biologically active PAF102 peptide can be produced quickly and efficiently in *P. pastoris* using the rice oleosin protein as a carrier to LDs.

Our results suggest that the oleosin fusion technology can be transferred to yeast cells for the production of recombinant proteins other than the AMPs here investigated. Particularly, we also successfully produced the GFP as an Ole18 fusion protein in *P. pastoris.* The main advantage of yeast over plant systems is the reduced production time. Oleosin technology in plants requires completion of the plant life cycle, since LDs are mainly accumulated in seeds: depending on the species, this could take several months whereas production in *P. pastoris* only requires several days. The production of other proteins should be evaluated to determine the robustness of the developed system.

## Data Availability

All datasets generated for this study are included in the manuscript and/or the Supplementary Files.

## Author Contributions

MC conceived and designed this study. PF contributed to the design. CP and TR prepared the gene constructs and carried out the yeast transformation experiments. CP and XS characterized the yeast transformants and the accumulation of AMPs in yeast cells. CP set up the LD isolation protocol. XS set up the recovery and evaluation of PAF102 from LDs. MC coordinated the study and prepared the manuscript. All authors read and approved the final manuscript.

## Conflict of Interest Statement

The authors declare that the research was conducted in the absence of any commercial or financial relationships that could be construed as a potential conflict of interest.

## References

[B1] Abell B. M. Holbrook L. A. Abenes M. Murphy D. J. Hills M. J. Moloney M. M. (1997). Role of the proline knot motif in oleosin endoplasmic reticulum topology and oil body targeting. *Plant Cell* 9 1481–1493. 10.1105/tpc.9.8.1481 9286116 PMC157013

[B2] Adelantado N. (2016). Lipidomics Studies of Recombinant Pichia pastoris for Improved Recombinant Protein Secretion Through Cell Engineering. Doctoral thesis Available at: https://www.tdx.cat/handle/10803/384229

[B3] Ahmad M. Hirz M. Pichler H. (2014). Protein expression in *Pichia pastoris*: recent achievements and perspectives for heterologous protein production. *Appl. Microbiol. Biotechnol.* 98 5301–5317. 10.1007/s00253-014-5732-5 24743983 PMC4047484

[B4] Aymé L. Baud S. Dubreucq B. Joffre F. Chardot T. (2014). Function and localization of the *Arabidopsis thaliana* diacylglycerol acyltransferase DGAT2 expressed in yeast. *PLoS One* 9:e92237. 10.1371/journal.pone.0092237 24663078 PMC3963872

[B5] Badosa E. Ferre R. Planas M. Feliu L. Besalú E. Cabrefiga J. (2007). A library of linear undecapeptides with bactericidal activity against phytopathogenic bacteria. *Peptides* 28 2276–2285. 10.1016/j.peptides.2007.09.010 17980935

[B6] Badosa E. Moiset G. Montesinos L. Talleda M. Bardají E. Feliu L. (2013). Derivatives of the antimicrobial peptide BP100 for expression in plant systems. *PLoS One* 8:e85515. 10.1371/journal.pone.0085515 24376887 PMC3871672

[B7] Beaudoin F. Wilkinson B. M. Stirling C. J. Napier J. A. (2000). In vivo targeting of a sunflower oil body protein in yeast secretory (sec) mutants. *Plant J.* 23 159–170. 10.1046/j.1365-313x.2000.00769.x 10929110

[B8] Bland J. M. Lucca A. J. D. (1998). Identification of cecropin a proteolytic cleavage sites resulting from *Aspergillus flavus* extracellular protease (s). *J. Agric. Food Chem.* 46 5324–5327. 10.1021/jf980638i

[B9] Boothe J. Nykiforuk C. Shen Y. Zaplachinski S. Szarka S. Kuhlman P. (2010). Seed-based expression systems for plant molecular farming. *Plant Biotechnol. J.* 8 588–606. 10.1111/j.1467-7652.2010.00511.x 20500681

[B10] Bouvier-Navé P. Benveniste P. Oelkers P. Sturley S. L. Schaller H. (2000). Expression in yeast and tobacco of plant cDNAs encoding acyl CoA: diacylglycerol acyltransferase. *Eur. J. Biochem.* 267 85–96. 10.1046/j.1432-1327.2000.00961.x 10601854

[B11] Bryksa B. C. Macdonald L. D. Patrzykat A. Douglas S. E. Mattatall N. R. (2006). A C-terminal glycine suppresses production of pleurocidin as a fusion peptide in *Escherichia coli*. *Protein Expr. Purif.* 45 88–98. 10.1016/j.pep.2005.04.010 15935695

[B12] Bundó M. Montesinos L. Izquierdo E. Campo S. Mieulet D. Guiderdoni E. (2014). Production of cecropin A antimicrobial peptide in rice seed endosperm. *BMC Plant Biol.* 14:102. 10.1186/1471-2229-14-102 24755305 PMC4032361

[B13] Bundó M. Shi X. Vernet M. Marcos J. F. López-García B. Coca M. (2019). Rice seeds as biofactories of rationally-designed and cell-penetrating antifungal PAF peptides. *Front. Plant Sci.* 10:731. 10.3389/fpls.2019.00731 31231409 PMC6566136

[B14] Cavallarin L. Andreu D. San Segundo B. (1998). Cecropin a—derived peptides are potent inhibitors of fungal plant pathogens. *Mol. Plant Microbe Interact.* 11 218–227. 10.1094/mpmi.1998.11.3.218 9487696

[B15] Chapman K. D. Dyer J. M. Mullen R. T. (2012). Biogenesis and functions of lipid droplets in plants: thematic review series: lipid droplet synthesis and metabolism: from yeast to man. *J. Lipid Res.* 53 215–226. 10.1194/jlr.R021436 22045929 PMC3269164

[B16] Coca M. Peñas G. Gómez J. Campo S. Bortolotti C. Messeguer J. (2006). Enhanced resistance to the rice blast fungus Magnaporthe grisea conferred by expression of a cecropin A gene in transgenic rice. *Planta* 223 392–406. 10.1007/s00425-005-0069-z 16240149

[B17] Ghosh C. Sarkar P. Issa R. Haldar J. (2019). Alternatives to conventional antibiotics in the era of antimicrobial resistance. *Trends Microbiol.* 27 323–338. 10.1016/j.tim.2018.12.010 30683453

[B18] Greenspan P. Mayer E. P. Fowler S. D. (1985). Nile red a selective fluorescent stain for intracellular lipid droplets. *J. Cell Biol.* 100 965–973. 10.1083/jcb.100.3.965 3972906 PMC2113505

[B19] Greer M. S. Truksa M. Deng W. Lung S. Chen G. Weselake R. J. (2015). Engineering increased triacylglycerol accumulation in Saccharomyces cerevisiae using a modified type 1 plant diacylglycerol acyltransferase. *Appl. Microbiol. Biotechnol.* 99 2243–2253. 10.1007/s00253-014-6284-4 25520169

[B20] Hancock R. E. W. Sahl H. G. (2006). Antimicrobial and host-defense peptides as new anti-infective therapeutic strategies. *Nat. Biotechnol.* 24 1551–1557. 10.1038/nbt1267 17160061

[B21] Ivashov V. A. Grillitsch K. Koefeler H. Leitner E. Baeumlisberger D. Karas M. (2013). Lipidome and proteome of lipid droplets from the methylotrophic yeast *Pichia pastoris*. *Biochim. Biophys. Acta* 1831 282–290. 10.1016/j.bbalip.2012.09.017 23041514 PMC3787741

[B22] Jacquier N. Mishra S. Choudhary V. Schneiter R. (2013). Expression of oleosin and perilipins in yeast promotes formation of lipid droplets from the endoplasmic reticulum. *J. Cell Sci.* 126 5198–5209. 10.1242/jcs.131896 24006263

[B23] Jamme F. Vindigni J. Méchin V. Cherifi T. Chardot T. (2013). Single cell synchrotron FT-IR microspectroscopy reveals a link between neutral lipid and storage carbohydrate fluxes in S. *cerevisiae*. *PLoS One* 8:e74421. 10.1371/journal.pone.0074421 24040242 PMC3770668

[B24] Jin F. Xu X. Zhang W. Gu D. (2006). Expression and characterization of a house X y cecropin gene in the methylotrophic yeast, *Pichia pastoris*. *Protein Expr. Purif.* 49 39–46. 10.1016/j.pep.2006.03.008 16647861

[B25] Jones K. H. Senft J. A. (1985). An improved method to determine cell viability by simultaneous staining with fluorescein diacetate-propidium iodide. *J. Histochem. Cytochem.* 33 77–79. 10.1177/33.1.2578146 2578146

[B26] Kang H. K. Kim C. Seo C. H. Park Y. (2017). The therapeutic applications of antimicrobial peptides (AMPs): a patent review. *J. Microbiol.* 55 1–12. 10.1007/s12275-017-6452-1 28035594

[B27] Koch B. Schmidt C. Daum G. (2014). Storage lipids of yeasts: a survey of nonpolar lipid metabolism. *FEMS Microbiol. Rev.* 38 892–915. 10.1111/1574-6976.12069 24597968

[B28] Laemmli U. K. (1970). Cleavage of structural proteins during the assembly of the head of bacteriophage T4. *Nature* 227 680–685. 10.1038/227680a0 5432063

[B29] Liu Q. Siloto R. M. P. Lehner R. Stone S. J. Weselake R. J. (2012). Acyl-CoA: diacylglycerol acyltransferase: molecular biology, biochemistry and biotechnology. *Prog. Lipid Res.* 51 350–377. 10.1016/j.plipres.2012.06.001 22705711

[B30] López-García B. Harries E. Carmona L. Campos-Soriano L. López J. J. Manzanares P. (2015). Concatemerization increases the inhibitory activity of short, cell-penetrating, cationic and tryptophan-rich antifungal peptides. *Appl. Microbiol. Biotechnol.* 99 8012–8021. 10.1007/s00253-015-6541-1 25846331

[B31] López-García B. Pérez-Payá E. Marcos J. F. (2002). Identification of novel hexapeptides bioactive against phytopathogenic fungi through screening of a synthetic peptide combinatorial library. *Appl. Environ. Microbiol.* 68 2453–2460. 10.1128/AEM.68.5.2453 11976121 PMC127571

[B32] López-García B. San Segundo B. Coca M. (2012). “Antimicrobial peptides as a promising alternative for plant disease protection,” in *Small Wonders: Peptides for Disease Control*, eds Rajasekarna K. Cary J. W. Jaynes J. Montesinos E. (Washintong, DC: ACS Books), 263–294. 10.1021/bk-2012-1095.ch013

[B33] Mannik J. Meyers A. Dalhaimer P. (2014). Isolation of cellular lipid droplets: two purification techniques starting from yeast cells and human placentas. *J. Vis. Exp.* 86 1–10. 10.3791/50981 24747783 PMC4160924

[B34] Marcos J. F. Muñoz A. Pérez-Payá E. Misra S. López-García B. (2008). Identification and rational design of novel antimicrobial peptides for plant protection. *Annu. Rev. Phytopathol.* 46 273–301. 10.1146/annurev.phyto.121307.094843 18439131

[B35] Montesinos E. Badosa E. Cabrefiga J. Planas M. Feliu L. Bardají E. (2012). “Antimicrobial peptides for plant disease control. From Discovery to Application,” in *Small Wonders: Peptides for Disease Control*, eds Rajasekaran K. Cary J. W. Jaynes J. Montesinos E. (Washintong, DC: ACS Books), 235–261. 10.1021/bk-2012-1095.ch012

[B36] Montesinos L. Bundó M. Badosa E. San Segundo B. Coca M. Montesinos E. (2017). Production of BP178, a derivative of the synthetic antibacterial peptide BP100, in the rice seed endosperm. *BMC Plant Biol.* 17:63. 10.1186/s12870-017-1011-9 28292258 PMC5351061

[B37] Montesinos L. Bundó M. Izquierdo E. Campo S. Badosa E. (2016). Production of biologically active cecropin a peptide in rice seed oil bodies. *PLoS One* 11:e0146919. 10.1371/journal.pone.0146919 26760761 PMC4711921

[B38] Moreno A. B. Del Pozo A. M. Borja M. Segundo B. S. (2003). Activity of the antifungal protein from *Aspergillus giganteus* against *Botrytis cinerea*. *Phytopathology* 93 1344–1353. 10.1094/PHYTO.2003.93.11.1344 18944061

[B39] Muñoz A. M. U. Read N. D. Marcos J. F. (2013). Understanding the mechanism of action of cell-penetrating antifungal peptides using the rationally designed hexapeptide PAF26 as a model. *Fungal Biol. Rev.* 26 146–155. 10.1016/j.fbr.2012.10.003

[B40] Nicolas P. (2009). Multifunctional host defense peptides: intracellular-targeting antimicrobial peptides. *FEBS J.* 276 6483–6496. 10.1111/j.1742-4658.2009.07359.x 19817856

[B41] Nykiforuk C. L. Boothe J. G. Murray E. W. Keon R. G. Goren H. J. Markley N. A. (2006). Transgenic expression and recovery of biologically active recombinant human insulin from *Arabidopsis thaliana* seeds. *Plant Biotechnol. J.* 4 77–85. 10.1111/j.1467-7652.2005.00159.x 17177787

[B42] Nykiforuk C. L. Shen Y. Murray E. W. Boothe J. G. Busseuil D. Rhéaume E. (2011). Expression and recovery of biologically active recombinant apolipoprotein AIMilano from transgenic safflower (Carthamus tinctorius) seeds. *Plant Biotechnol. J.* 9 250–263. 10.1111/j.1467-7652.2010.00546.x 20618764

[B43] Paltauf F. Daum G. Zellnig G. Leber R. Zinser E. (1994). Characterization of lipid particles of the yeast,Saccharomyces cerevisiae. *Yeast* 10 1421–1428. 10.1002/yea.320101105 7871881

[B44] Parmenter D. L. Boothe J. G. van Rooijen G. J. Yeung E. C. Moloney M. M. (1995). Production of biologically active hirudin in plant seeds using oleosin partitioning. *Plant Mol. Biol.* 29 1167–1180. 10.1007/BF00020460 8616216

[B45] Parthibane V. Rajakumari S. Venkateshwari V. Iyappan R. Rajasekharan R. (2012). Oleosin is bifunctional enzyme that has both monoacylglycerol acyltransferase and phospholipase activities. *J. Biol. Chem.* 287 1946–1954. 10.1074/jbc.M111.309955 22128159 PMC3265875

[B46] Peschel A. Sahl H.-G. (2006). The co-evolution of host cationic antimicrobial peptides and microbial resistance. *Nat. Rev. Microbiol.* 4 529–536. 10.1038/nrmicro1441 16778838

[B47] Popa C. Li L. Gil S. Tatjer L. Hashii K. Tabuchi M. (2016). The effector AWR5 from the plant pathogen *Ralstonia solanacearum* is an inhibitor of the TOR signalling pathway. *Sci. Rep.* 6:27058. 10.1038/srep27058 27257085 PMC4891724

[B48] Schägger H. von Jagow G. (1987). Tricine-sodium dodecyl sulfate-polyacrylamide gel electrophoresis for the separation of proteins in the range from 1 to 100 kDa. *Anal. Biochem.* 166 368–379. 10.1016/0003-2697(87)90587-2 2449095

[B49] Sierra J. M. Fusté E. Rabanal F. Vinuesa T. Viñas M. (2017). An overview of antimicrobial peptides and the latest advances in their development. *Expert Opin. Biol. Ther.* 17 663–676. 10.1080/14712598.2017.1315402 28368216

[B50] Steiner H. Hultmark D. Engström A. Bennich H. Boman H. G. (1981). Sequence and specificity of two antibacterial proteins involved in insect immunity. *Nature* 292 246–248. 10.1038/292246a0 7019715

[B51] Suttmann H. Retz M. Paulsen F. Harder J. Zwergel U. Kamradt J. (2008). Antimicrobial peptides of the Cecropin-family show potent antitumor activity against bladder cancer cells. *BMC Urol.* 8:5. 10.1186/1471-2490-8-5 18315881 PMC2276511

[B52] Ting J. T. L. Balsamo R. A. Ratnayake C. Huang A. H. C. (1997). Oleosin of plant seed oil bodies is correctly targeted to the lipid bodies in transformed yeast. *J. Biol. Chem.* 272 3699–3706. 10.1074/jbc.272.6.3699 9013626

[B53] Tzen J. T. C. (2012). Integral proteins in plant oil bodies. *ISRN Bot.* 2012 1–16. 10.5402/2012/173954

[B54] Valore E. V. Ganz T. (1997). “Laboratory Production of Antimicrobial Peptides in Native Conformation,” in *Antibacterial Peptide Protocols*, ed. Shafer W. M. (Totowa, NJ: Humana Press), 115–131. 10.1385/0-89603-408-9:115 9276301

[B55] van Rooijen G. J. Moloney M. M. (1995). Plant seed oil-bodies as carriers for foreign proteins. *BioTechology* 13 72–77. 10.1038/nbt0195-72 9634752

[B56] Vindigni J. Wien F. Giuliani A. Erpapazoglou Z. Tache R. Jagic F. (2013). Fold of an oleosin targeted to cellular oil bodies. *Biochim. Biophys. Acta* 1828 1881–1888. 10.1016/j.bbamem.2013.04.009 23603223

[B57] Vismer H. F. Shephard G. S. Westhuizen L. Van Der Mngqawa P. Bushula-njah V. Leslie J. F. (2019). Mycotoxins produced by Fusarium proliferatum and F. Pseudonygamai on maize, sorghum and pearl millet grains in vitro. *Int. J. Food Microbiol.* 296 31–36. 10.1016/j.ijfoodmicro.2019.02.016 30826540

[B58] Wang M. Zheng K. Lin J. Huang M. Ma Y. Li S. (2018). Rapid and efficient production of cecropin A antibacterial peptide in *Escherichia coli* by fusion with a self-aggregating protein. *BMC Biotechnol.* 18:62. 10.1186/s12896-018-0473-7 30290795 PMC6173929

[B59] Yu G. Baeder D. Y. Regoes R. R. Rolff J. (2018). Predicting drug resistance evolution: insights from antimicrobial peptides and antibiotics. *Proc. R. Soc.* 285:20172687. 10.1098/rspb.2017.2687 PMC5879628 29540517

[B60] Zasloff M. (2002). Antimicrobial peptides of multicellular organisms. *Nature* 415 389–395. 10.1038/415389a 11807545

[B61] Zhang L. Gallo R. L. (2016). Antimicrobial peptides. *Curr. Biol.* 26 81–95. 10.1007/978-3-319-29785-9_6 26766224

