# Supplementary material for: Biotechnological Production of the Cell Penetrating Antifungal PAF102 Peptide in Pichia pastoris
Source: Front Microbiol. 2019 Jun 27;10:1472. doi: 10.3389/fmicb.2019.01472 (PMC6610294; doi:10.3389/fmicb.2019.01472)
Supplement: Supplementary file 1 [file Table_1.DOCX]

**Supplementary Table 1.** Primers used in this study

| EcoRI_Ole18fwd | 5'CG**GAATTC**ATGGCGGATCGCGACCGCGC3' |  |
| --- | --- | --- |
| Ole18rev_wo stop | 5'CTTTACTCATTGGCGAGGATGTCTTGGTGCC3' |  |
| GFPfwd | 5'GACATCCTCGCCAATGAGTAAAGGAGAA3' |  |
| XbaI_GFPrev | 5'CC**TCTAGA**TTATTTGTATAGTTCATCCATGCCATGT3' |  |
| XhoI_Ole18rev_with stop | 5'CG**CTCGAG**TTACGAGGATGTCTTGGTGC3' |  |
| XhoI_CecArev | 5'CG**CTCGAG**TTATCACTTGGCGATTTGGG3' |  |
| XhoI_PAF102rev | 5'CG**CTCGAG**TTACTACCAGAACCACTTCT3' |  |
| Dgat1_fwd | 5'GGCCGTCTCGGATCGGTACCATGGCGATTTTGGATTCTG3' |  |
| Dgat1_rev | 5'TTCTGAGATGAGTTTTTGTTTCATGACATCGATCCTTTTC3' |  |
